# Integrative Analysis of Pharmacokinetic and Metabolomic Profiles for Predicting Metabolic Phenotype and Drug Exposure Caused by Sotorasib in Rats

**DOI:** 10.3389/fonc.2022.778035

**Published:** 2022-04-05

**Authors:** Ping Du, Lihong Liu, Ting Hu, Zhuoling An

**Affiliations:** Department of Pharmacy, Research Unit, Beijing Chao-Yang Hospital, Capital Medical University, Beijing, China

**Keywords:** sotorasib, pharmacokinetics, pharmacometabolomics, integrative analysis, HPLC-MS/MS

## Abstract

Sotorasib is a novel targeted inhibitor of Kirsten rat sarcoma (KRAS) (G12C) that has shown exciting tumor-suppressing effects not only for single targeted agents but also for combination with immune checkpoint inhibitors. However, no integrative analysis of the pharmacokinetics (PK) and pharmacometabolomics (PM) of sotorasib has been reported to date. In the present study, a sensitive and robust high-performance liquid chromatography–tandem mass spectrometry (HPLC-MS/MS) method was firstly developed and fully validated for the quantitation of sotorasib in rat plasma. After one-step protein precipitation, sotorasib and an internal standard (carbamazepine) were separated on a Waters XBrige C_18_ column (50 mm × 2.1 mm, 3.5 μm) and analyzed in electrospray ionization positive ion (ESI^+^) mode. The optimized method was fully validated according to guidance and was successfully applied for the PK study of sotorasib at a dose of 10 mg/kg. In addition, a longitudinal and transversal PM was employed and correlated with PK using partial least squares model and Pearson’s analysis. With multivariate statistical analysis, the selected six (AUC model) and nine (*C*_max_ model) metabolites completely distinguished the high- and low-exposure groups after sotorasib treatment, which indicates that these potential biomarkers can predict drug exposure or toxicity. The results of this study will not only shed light on how sotorasib disturbs the metabolic profiles and the relationship between PK and PM but also offer meaningful references for precision therapy in patients with the KRAS (G12C) mutation.

## 1 Introduction

Cancer, which has increased incidence and mortality rates, has become one of the chronic diseases that seriously endanger human health. According to the latest literature and statistics, there are 19.3 million new cancer cases worldwide and 10 million deaths in 2020 (1). Mutation in Kirsten rat sarcoma (KRAS) viral oncogene homolog, which can potentiate the tumor-promoting activity, is one of the most common carcinogenic events in endodermal cancer (2). In fact, KRAS mutations have been identified predominantly in lung (approximately 25% of cases), pancreatic (about 95% of case), and colorectal (approximately 35% of cases) cancers (3). In KRAS mutated tumors, 80% of the carcinogenic mutations occur in codon 12, and the most popular mutation sites are KRAS (G12D), KRAS (G12V), and KRAS (G12C) (4). The following characteristics of KRAS have led to many challenges in its druggability development: 1) KRAS binds to guanosine diphosphate (GDP) and guanosine triphosphate (GTP) with picomolar affinity, which seriously hinders the development of nucleotide competitive inhibitors, and 2) the KRAS protein lacks ideal small-molecule binding pockets, which makes it difficult to design high-affinity allosteric inhibitors (5). However, when KRAS (G12C) is in an inactive GDP-bound state, the structure-based optimization inhibitor is covalently bound to the mutant cysteine residue and holds a pocket in the switch II region (SIIP). Due to its excellent antitumor efficacy in preclinical analyses, sotorasib, a novel targeted inhibitor of KRAS (G12C), precisely blocks the KRAS (G12C) inactive GDP-bound state. Additionally, sotorasib has shown exciting tumor-suppressing effects not only for single targeted agents but also for combination with immune checkpoint inhibitors (6). At present, based on previous clinical trials (7–9), sotorasib has been approved by the US Food and Drug Administration (FDA) for the treatment of locally advanced/metastatic KRAS (G12C) solid tumors (10).

It is well known that comprehensive understanding of the pharmacokinetics (PK), pharmacodynamics (PD), and pharmacometabolomics (PM) is an indispensable element for further improving the risk–benefit ratio of patients. Despite the high-performance liquid chromatography–tandem mass spectrometry (HPLC-MS/MS) methods published by Madhyastha et al. (11) and Retmana et al. (12) for the quantification of sotorasib in mouse plasma and tissue homogenates, a wider linear range (1.08–5,040 and 2–2,000 ng/ml, respectively), a higher lower limit of quantitation (LLOQ; 1.08 and 2 ng/ml, respectively), and the laborious sample extraction (liquid–liquid extraction coupled with evaporation and three-step protein precipitation, respectively) will decrease the quantitative throughput and efficiency to some extent, especially for large preclinical and clinical sample sizes. Undoubtedly, the detection sensitivity of the reported method still has much room for improvement. However, as far as we know, no HPLC-MS/MS analysis or PK has been delivered for the rapid and robust quantitation of sotorasib in rat plasma. Therefore, taking into account the urgency of cancer therapy and the emerging need for preclinical and clinical blood concentrations/exposures or robust analytical methods, a reliable and reproducible HPLC-MS/MS assay was developed, validated, and successfully applied for the quantitation of sotorasib in rat plasma. The results may provide a significant mirror for future KRAS-targeted therapy, PK investigation, and therapeutic drug monitoring in preclinical or clinical studies/trials.

Metabolomics is one of the most powerful tools used to investigate the interaction between the genetic background and the exogenous and endogenous factors of human health (13). The concept of PM was firstly illustrated in a study which demonstrated that metabolomic information in drug-free urine samples is predictive of both drug metabolism and the toxicity of paracetamol (14). Indeed, PM can not only reveal the terminal metabolic profile by drug treatment but also reflect the metabolic status between tissues and fluids, which will be beneficial to understanding the biological mechanisms of diseases or drugs (15, 16). Unfortunately, to the best of our knowledge, no comprehensive metabolic profiling studies pertaining to sotorasib treatment both *in vitro* and *in vivo*, especially with regard to metabolite reprogramming/perturbation caused by drug treatment, have been reported.

In view of the shortcomings described above, the objective of this study was originally proposed as to longitudinally and transversally illustrate the metabolic fingerprint induced by sotorasib in rats using our previously reported HPLC-MS/MS method (17, 18). Furthermore, by means of several multivariate statistical analyses, an integrative analysis of PM and PK was employed in order to predict the metabolic phenotype and drug exposure. Overall, we will reveal the first in-depth interrogation of trajectory changes that benefit propitious understanding of how sotorasib interacts with small molecular metabolites. Moreover, several candidate predictive biomarkers were investigated and validated for drug response or toxicity. The results of this study will not only shed light on how sotorasib disturbs the metabolic profiles and the relationship between PK and PM but also offer meaningful references for precision therapy in patients with the KRAS (G12C) mutation.

## 2 Materials and Methods

### 2.1 Chemicals

Sotorasib (lot: DC2229802; purity, ≥98%) was purchased from DC Chemicals Company (Shanghai, China). The internal standard (IS) (carbamazepine; purity, ≥99%) was obtained from the National Institutes for Food and Drug Control (Beijing, China). All standards of metabolites and stable isotope-labeled internal standards (ILIS) were obtained from the following companies: Sigma-Aldrich (St. Louis, MO, USA), Cayman Chemical (Ann Arbor, MI, USA), Bidepharm (Shanghai, China), Steraloids (Newport, RI, USA), and Cambridge Isotope Laboratories (Andover, MA, USA) (**Supplementary Table S1**). Detailed information is provided in a previous work (18). Methanol (MeOH), acetonitrile (ACN), isopropanol (IPA), and formic acid were of high-performance liquid chromatography (HPLC) grade and were utilized to prepare stock or working solutions, mobile phases. Drug-free heparinized plasma was collected from healthy rats. Ultrapure water was produced using the Milli-Q reference water purification system.

### 2.2 Analytical Instruments and HPLC-MS/MS Conditions

For the PK study, a chromatographic system of Shimadzu LC-20ADXR (Kyoto, Japan) coupled with a mass spectrometer (QTRAP 5500; SCIEX, Concord, Canada) was utilized for chromatographic separation and mass quantitation (19). The column temperature was set to 40°C on a reversed-phase C_18_ column (50 mm × 2.1 mm i.d., 3.5 μm) (XBrige; Waters, Milford, MA, USA), and the total run time was 3.0 min. Both mobile phases contained 0.1% formic acid. Gradient elution was adopted to comply with the compounds’ baseline separation (0–0.1 min, 90% water phase; 0.1–1.2 min, 10% water phase; 1.2–2.2 min, 0% water phase; and 2.2–3.0 min, 90% water phase). The flow rate was fixed at 0.4 ml/min with an injection volume of 2 μl. A time-efficient washing procedure composed of MeOH/water/ACN/IPA (1:1:1:1, *v/v/v/v*) was applied during the entire analyses. Multiple reaction monitoring (MRM) was utilized with the following parameters: ion spray voltage, 5,500 V; temperature, 550°C; curtain gas, 20.0 psi; ion source, gas 1 at 55.0 psi; and turbo ion source, gas 2 at 55.0 psi. A proton adduct [M+H]^+^ ion and double MRM transitions were used, which were set at *m/z* 561.4→317.1 (qualifier) and *m/z* 561.4→134.1 (quantifier) for sotorasib and at 237.0→194.1 for the IS.

For metabolic profiling analysis, the HPLC-MS/MS system (Spark Holland; API 5500, SCIEX, Concord, Canada) was adopted for targeted metabolomic analysis. The chromatography columns (Waters BEH, HSS T3) and elution solvent (gradient elution) were all evaluated and employed according to our previous study (18). The column temperature was set at 20°C with an injection volume of 5 μl. The water and organic phases (ACN/IPA = 7:2, *v/v*) contained 0.1% formic acid, and gradient elution was achieved within 27 min. The detailed optimized MRM parameters are shown in **Supplementary Table S1** and in a previous study (18).

### 2.3 Stock and Working Solutions, Calibration Standards, and Quality Control Samples

Stock solutions of sotorasib and IS were equipped using ACN at a concentration of 1.0 mg/ml and further diluted for the calibration standards and quality control (QC) samples at a final concentration of 500 µg/ml separately. The working IS solution was prepared in MeOH at a concentration of 10 ng/ml. The working calibration solutions of sotorasib were completed in dilution (MeOH/water = 1:1, *v/v*) to obtain concentrations of 5, 10, 20, 50, 100, 100, 200, 500, 1,000, 2,000, and 5,000 ng/ml. Similarly, the QC working solutions of analytes were prepared to obtain final concentrations of 10, 200, and 4,000 ng/ml. All solutions were stored in a −80°C freezer pending analysis. For the PK study, calibrations were made in blank rat plasma to achieve a nominal concentration range of 0.5–500 ng/ml. QC plasma samples were prepared in blank rat plasma at nominal concentrations of 1, 20, and 400 ng/ml.

Additionally, pooled standard solutions of all metabolites and ILIS were prepared for the calibration curve (0.2–5,000 ng/ml) in this PM study. To ensure reliable quantitation of all analytes and better comparability in routine analysis, plasma QC samples were produced by mixing equal volumes of unknown plasma from all the unknown plasma samples. Briefly, six aliquots of pooled QCs were constructed as actual and interpolated the analytical sequence to test the status of the HPLC-MS/MS system.

### 2.4 Sample Preparation

During the PK study, all unknown plasma samples were mixed for 1 min and 20 μl aliquot of plasma was pipetted into an Eppendorf centrifuge tube. Protein precipitation was adopted for the preparation of the sample. After the addition of 10 μl IS working solution (10 ng/ml) and 80 μl MeOH, proteins were extracted by vortex mixing for approximately 30 s. The supernatant was centrifuged at 13,500 rpm for 10 min at 4°C. The extraction solutions were then pipetted into autosampler vials.

For the analysis of the metabolic characteristics, a one-step protein precipitation method was also employed. Briefly, an aliquot of 50 μl plasma was added with 10 μl IS mixture (eight ISs, 400 ng/ml) and 140 μl precipitation solution (−20°C methanol). Afterward, the mixture was vortexed for 2 min and centrifuged (13,500 rpm, 10 min at 4°C). The upper solutions were injected into the HPLC-MS/MS system for analysis.

### 2.5 Bioanalytical Method Validation for Pharmacokinetics

Validation of the biological method was executed in line with the guidelines issued by the FDA (20) and the Chinese Pharmacopeia (version 2015) (21).

To evaluate the specificity and selectivity, six individual heparin-anticoagulated rat plasma samples were processed. Each sample was prepared as double blank (neither analytes nor IS), blank (only IS), LLOQ, and unknown rat plasma. The chromatographic integrity and possible disturbance were compared.

Sensitivity analysis was carried out in the form of LLOQ, which is the lowest concentration with accuracy and precision of less than or equal to ±20% and a signal-to-noise (S/N) ratio higher than 5:1. To evaluate the carryover, three consecutive double-blank samples were injected following the upper limit of quantification (ULOQ) standard.

With regard to the intra- and inter-accuracy and precision, the LLOQ (0.5 ng/ml), low QC (LQC, 1 ng/ml), medium QC (MQC, 20 ng/ml), and high QC (HQC, 400 ng/ml) were set and assessed (*n* = 6). Calibration standard weighted linear regression (1/*x* ^2^) was carried out in duplicate. The calibration curves were defined by the ratio of the peak area of the analyte (sotorasib) and IS (19).

The recovery of sotorasib and IS was investigated by comparing the peak areas of each sotorasib and IS at three QC levels, where plasma was added with the analyte before and after the extraction (19).

Matrix effect (ME) was evaluated at QC levels in six replicates by comparing their responses to those of the reference solutions without the presence of any matrix at QC levels. The corresponding peak area ratios of the analyte to IS in spiked plasma post-extraction (*A*) were then compared with those of the water-substituted samples (*B*) at equivalent concentrations. This ratio (*A*/*B* × 100) is defined as ME. The MEs of IS were determined in a similar manner. The criterion for the acceptability of the data was the inter-subject variability range within 15% (19).

Stability was investigated at LQC and HQC concentrations in six replicates. The stability conditions incorporated the short-term (at ambient temperature for 24 h), freeze–thaw (−80°C to ambient temperature, three sequential cycles), post-preparation (autosampler, 24 h), and long-term (−80°C for 3 months) status. If the %Bias is within ±15%, the stability is considered acceptable.

### 2.6 Pharmacokinetic Study and Incurred Sample Reanalysis

Six Sprague–Dawley rats (180–220 g, 8-week-old males; animal license: SCXK-2019-0008) were purchased from Beijing Vital River Laboratory Animal Technologies Co., Ltd. (Beijing, China). All rats were fed in a specific pathogen-free (SPF) conditioned room to allow acclimatization for 1 week. Animal experiments were implemented in line with the Guidelines for the Care and Use of Laboratory Animals, and animal ethics was approved by Beijing Chao-Yang Hospital, Capital Medical University. Rats were orally administered sotorasib dissolved with 1% Tween-80 and 2% hydroxypropyl methylcellulose (HPMC) in water at a dose of 10 mg/kg. Biological samples (approximately 500 μl) were collected from ophthalmic veins into heparin-anticoagulated collection tubes before administration and at 15 min, 30 min, and 1, 1.5, 2, 2.5, 3, 4, and 8 h after administration. Plasma samples were acquired by direct centrifugation at 3,500 rpm for 10 min at 4°C.

Incurred sample reanalysis (ISR) was carried out to critically support reliable determination using the HPLC-MS/MS method. Twelve incurred samples, which were from around *C*_max_ (maximum concentration) and in the elimination phase, were reanalyzed in separate batches. As for the acceptance criteria, the concentrations of the initial quantitation and reanalysis should be ≤20% of the mean values for at least two of the three repeats (20, 22).

### 2.7 Uncertainty of Measurement

It is noted that uncertainty of measurement (UM) is a meaningful indicator of robust and reliable quantitation (23). The bottom-up approach was utilized to evaluate the measurement uncertainty according to previous literature (23, 24). Both standard uncertainty and expanded uncertainty (*k* = 2, 95% confidence limits) were evaluated in light of the Guide to the Expression of Uncertainty in Measurement. The procedures for estimating measurement uncertainty require that the analysis process be traceable, determine the performance characteristics of the equipment, and determine the source and impact of the uncertainty. Several sources of uncertainty, such as volumetric operations, weighting, multiple-point calibration (linear regression), and recovery, were considered in the present work. Samples at a concentration of 20 ng/ml (*n* = 18) were analyzed to evaluate the measurement uncertainty of the HPLC-MS/MS method (22).

### 2.8 Pharmacometabolomics of Sotorasib

A total of 289 metabolites, which contained amino acids, bile acids, and vitamins, were included in the metabolomics method being considered. All biologically active metabolites can be quantitated within 27 min. The calculation linearity ranged from 0.2 to 5,000 ng/ml, which provided powerful capability for the successful quantitation of low-abundance compounds. For the longitudinal PM of sotorasib, the same plasma samples were utilized before (0 h, pre-dose) and after administration (post-dose, until 8 h). For the transversal PM, raw metabolomic data were divided into two parts: before (pre-dose) and after (post-dose) administration. The metabolomic profiling and trajectory effects of sotorasib were investigated and analyzed using multiple variable statistical analysis methods.

### 2.9 Multivariate Statistical Analysis and Data Processing

Raw data files were processed and checked using Analyst 1.6.3 and MultiQuant 3.0.1 (SCIEX, Concord, Canada). The concentrations of the analytes were calculated according to the calibration curve. Pharmacokinetic parameters, such as *C*_max_ and the area under the curve (AUC), were calculated using Phoenix WinNonLin software (Pharsight 8.3, Mountain View, CA, USA). Pearson’s correlation was employed to investigate the correlation between the metabolomic data and PK parameters using IBM SPSS 26.0 (Armonk, NY, USA). SIMCA-P software (v14.1; Umetric, Umeå, Sweden) was employed to build mathematical models, including unsupervised principal component analysis (PCA), supervised orthogonal projection to latent structures discriminant analysis (OPLS-DA), and partial least squares (PLS). Compounds with values of variable importance in the projections (VIP) >1 and statistical significance of *p* < 0.05 were selected for further identification and metabolic pathway analysis. One hundred or 200 random permutation tests were employed to examine overfitting and random effects, which can assess the predictive ability of the model. Meanwhile, significant variables with *p* < 0.05 and fold change (FC) >2 or <0.5 were considered potential biomarkers. Pathway analysis was achieved using online MetaboAnaylst 5.0 (http://www.metaboanalyst.ca). A *p*-value less than 0.05 was considered statistically significant.

## 3 Results

### 3.1 Method Development for the Quantitation of Sotorasib

**Figure 1:**
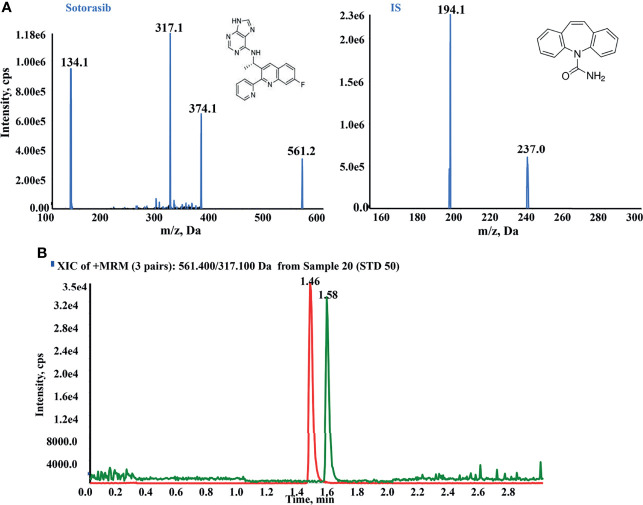
High-performance liquid chromatography–tandem mass spectrometry (HPLC-MS/MS) conditions for the quantitation of sotorasib. **(A)** Product ion mass spectra and chromatogram of sotorasib and the internal standard (IS; carbamazepine) in ESI^+^ mode. **(B)** Typical chromatogram of sotorasib (1.46 min) and IS (1.58 min) at the indicated retention times.

To optimize the mass spectrometric conditions, an injection pump was utilized to obtain the highest response of any analytes. Qualified and quantitative detection was performed using ESI^+^ and MRM. The product ion fragmentations are shown in **Figure 1A**. The most abundant and better separated product ions were identified for determination. Finally, baseline separation can be carried out, with retention times of 1.46 and 1.58 min for sotorasib and IS, respectively (**Figure 1B**).

### 3.2 Method Validation for the Quantitation of Sotorasib

#### 3.2.1 Selectivity and Specificity

**Figure 2:**
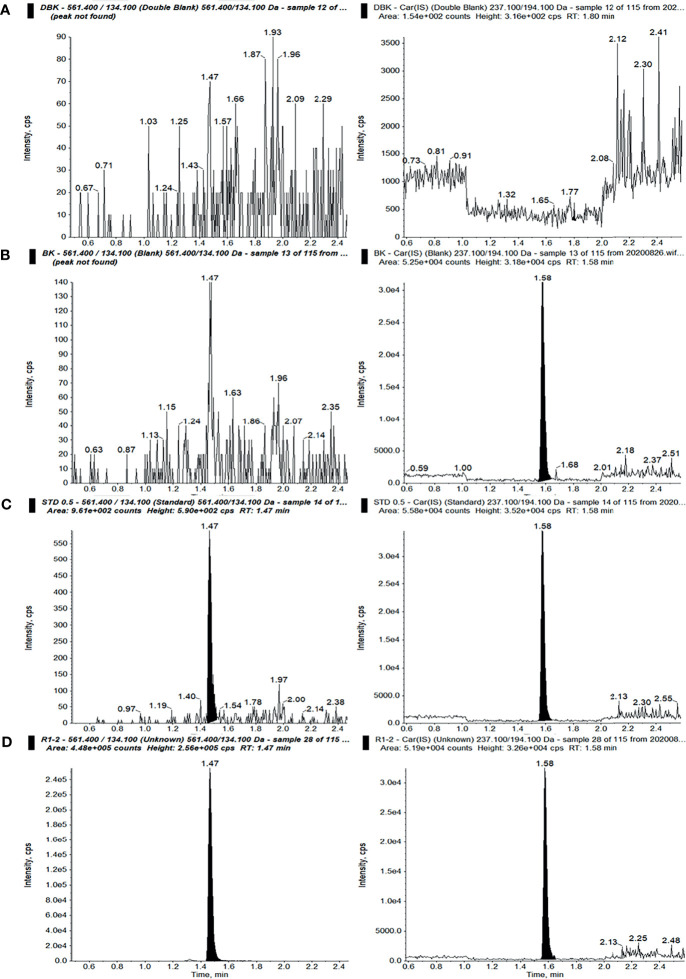
Ion chromatograms of sotorasib (*left*) and the internal standard (IS) (*right*) in double-blank plasma without analyte and IS **(A)**, blank plasma with IS **(B)**, blank plasma with sotorasib at the lower limit of quantitation (LLOQ) with IS **(C)**, and an unknown sample after administration of a 10-mg/kg dose of sotorasib **(D)** (298.36 ng/ml, 15 min after administration).

The selectivity and specificity results are an important assurance of the performance quality of the HPLC-MS/MS method. As shown in **Figure 2**, no interference peak was found under the present HPLC-MS/MS conditions at the indicated retention times, demonstrating the better selectivity and specificity of the employed method.

#### 3.2.2 Accuracy, Precision, Carryover, and Linearity

The accuracy and precision results (**Table 1**) indicated that a LLOQ of 0.5 ng/ml could meet the requirements of the test. Also, the limit of detection of sotorasib was 0.5 ng/ml at an S/N ratio ≥3. As listed in **Table 1**, the intra- and inter-day accuracy and precision values were all less than or equal to ±4.42% and 9.42%, respectively.

**Table 1:** Intra- and inter-day accuracy and precision of the HPLC-MS/MS method in rat plasma (*n* = 6).

Concentration (ng/ml)	Intra-day			Inter-day		
	Mean ± SD	CV (%)	Bias (%)	Mean ± SD	CV (%)	Bias (%)
0.5	0.50 ± 0.04	7.10	−0.57	0.51 ± 0.01	1.96	2.00
1	1.02 ± 0.10	9.42	1.59	0.98 ± 0.05	5.09	−1.80
20	20.42 ± 0.83	4.05	2.08	20.35 ± 0.61	3.00	1.75
400	410 ± 22.77	5.55	2.50	417.67 ± 7.79	1.87	4.42

CV, coefficient of variation.

During the validation procedure, carryover was evaluated. Finally, a washing/rinsing solution of MeOH/water (1:1, *v/v*) was selected to overcome carryover, and suitable accuracy and quantitative determination was achieved. No carryover was observed under the present conditions. A calibration curve was obtained within the range of 0.5–500 ng/ml with a correlation coefficient (*r*) of more than 0.990.

#### 3.2.3 Recovery, Matrix Effect, and Stability

Protein precipitation (PPT) is a common, high-throughput, and energy-efficient method for sample extraction. After step-by-step validation, a simple one-step PPT using an aliquot of 20 µl plasma was used for sample preparation. As shown in **Table 2**, the recovery results demonstrated that the mean recovery of sotorasib was consistently within the range of 78.20%–84.80% and with a CV of ≤6.54%. The range of the IS-normalized matrix effects was 123.35%–124.83%, with a CV of <0.10%, which proved the consistent matrix enhancement, to some extent. Besides, the accuracy/precision results also indicated that the PPT procedure could satisfy the requirements for the quantitative determination of sotorasib (**Table 1**).

**Table 2:** Recovery and matrix effects of sotorasib (*n* = 6).

Concentration (ng/ml)	Recovery (%)		Matrix effect (%)		IS-normalized matrix effect (%)	
	Mean ± SD	CV (%)	Mean ± SD	CV (%)	Mean ± SD	CV (%)
1	84.80 ± 4.58	5.40	143.02 ± 0.06	0.04	123.35 ± 0.10	0.08
20	81.43 ± 1.92	2.35	126.08 ± 0.07	0.06	124.12 ± 0.13	0.10
400	78.20 ± 5.11	6.54	127.45 ± 0.70	0.05	124.83 ± 0.13	0.10

CV, coefficient of variation; IS, internal standard.

Regarding stability, the results for the low and high QC samples are presented in **Table 3**. The bias and CV values were all within ±15%. Sotorasib was stable under several storage conditions in plasma or in solution, such as at ambient temperature for 6 h, freeze–thaw procedures for three cycles, post-preparation in the autosampler for 24 h, working solution at −80°C for 10 days, and long-term stability at −80°C for 3 months. Overall, the present stability results were consistent with those in previous studies (11, 12).

**Table 3:** Stability of sotorasib under different storage conditions (*n* = 6).

Concentration (ng/ml)	Room temperature stability		Post-preparation stability		Freeze–thaw stability		Working solution stability		Long-term stability	
	Mean ± SD	CV (%)	Mean ± SD	CV (%)	Mean ± SD	CV (%)	Mean ± SD	CV (%)	Mean ± SD	CV (%)
1	1.03 ± 0.10	4.70	1.07 ± 0.06	5.30	1.06 ± 0.07	6.45	1.034 ± 0.08	8.13	1.01 ± 0.07	5.30
400	434.00 ± 8.07	7.80	441.20 ± 10.76	2.44	448.60 ± 6.19	1.38	409.37 ± 37.98	9.28	399.67 ± 20.10	3.69

CV, coefficient of variation.

### 3.3 Uncertainty of Measurement

**Figure 3:**
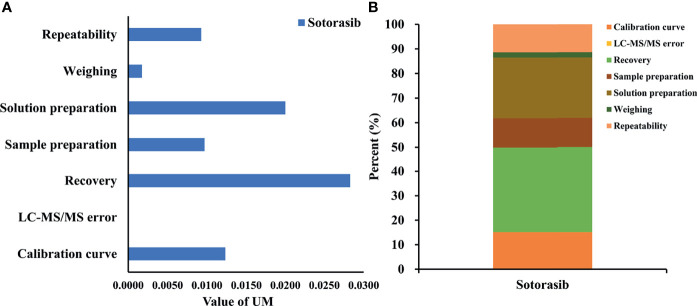
Uncertainty of measurement and proportion for determining sotorasib at a concentration of 20 ng/ml. **(A)** Statistical histogram of the uncertainty components. **(B)** The percentage of each element in the uncertainty components.

The results in **Figures 3A, B** showed that uncertainty of determination could be attributed to the uncertainty of repeatability, sample weighting, solution preparation, sample extraction and recovery, the HPLC-MS/MS system, and the calibration curve. The largest uncertainty at a concentration of 20 ng/ml was from recovery. The final expression of measurement uncertainty at a concentration of 20 ng/ml was 20.42 ± 8.06 ng/ml (mean value ± expanded uncertainty) for sotorasib with a coverage factor value of *k* = 2 (95% confidence limits). Taken together, when preparing sample extraction, attention should be paid to the state of plasma, the setting of the calibration range, and pretreatment of the biological sample to minimize its impact on the uncertainty (22).

### 3.4 Pharmacokinetics and Incurred Sample Reanalysis

**Figure 4:**
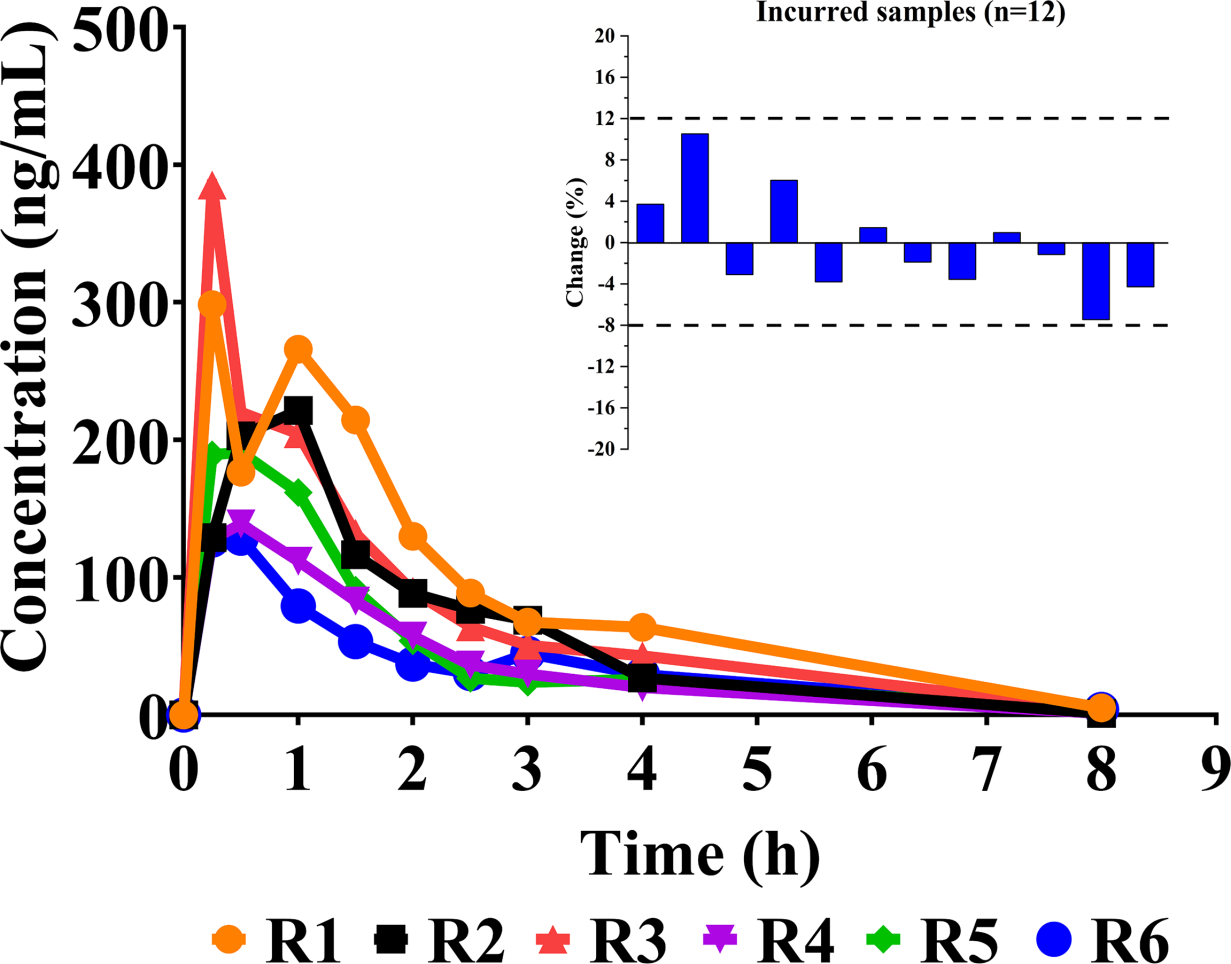
Individual concentration–time curves of sotorasib in rats after its oral administration at a dose of 10 mg/kg (*n* = 6). Also shown is a graphical representation of the results of incurred sample reanalysis (ISR).

After oral administration, sotorasib was rapidly metabolized. **Figure 4** describes the individual concentration–time profiles and the ISR. The summarized PK parameters are listed in **Table 4**. The results of PK were almost consistent with those of other reported studies at similar doses (11, 12).

**Table 4:** Pharmacokinetic parameters of sotorasib in rats (*n* = 6).

Parameters	Sotorasib
*t*_1/2_ (h)	1.24 ± 0.22
*C*_max_ (ng/ml)	227.28 ± 98.83
*T*_max_ (h)	0.5 (0.25–1)
AUC_0–_ *_t_* (ng·h/ml)	457.05 ± 165.17
AUC_0–∞_ (ng·h/ml)	462.75 ± 166.63
Vz_F (L/kg)	43.28 ± 17.90
CL_F (L h^−1^ kg^−1^)	24.00 ± 8.04
MRT (h)	1.89 ± 0.18

Values shown were the mean ± SD, except for T_max_, which is expressed as median (range).

C_max_, maximum concentration; T_max_, time to maximum; AUC, area under the curve; Vz_F, apparent volume of distribution; CL_F, apparent clearance; MRT, mean residence time.

### 3.5 Longitudinal and Transversal Metabolomics of Sotorasib

**Figure 5:**
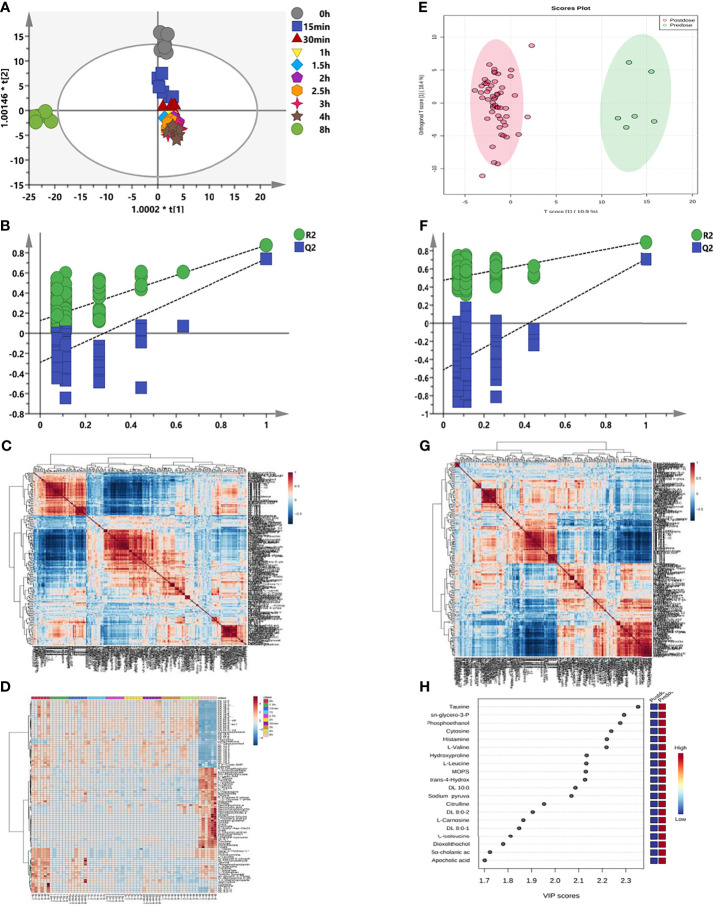
Longitudinal and transversal metabolic profiles of sotorasib. **(A)** Longitudinal orthogonal projection to latent structures discriminant analysis (OPLS-DA) score plot according to the different time points. **(B)** Random permutation test with 200 iterations from **(A)** for longitudinal metabolomics. **(C)** Longitudinal hierarchical cluster analysis (HCA) of the metabolite–metabolite correlations in response to sotorasib treatment. **(D)** Longitudinal heatmap clustering of the changed metabolites. **(E)** Transversal OPLS-DA plot. **(F)** Transversal permutation test with 200 iterations. **(G)** Transversal HCA correlation analysis before and after sotorasib treatment. **(H)** Transversal metabolomics for variable importance in the projections (VIP) values of the top 20 metabolites due to sotorasib treatment.

As shown in **Figure 5A**, the logitudinal metabolic characteristics of sotorasib were clustered according to the sampling time points, and the metabolic fingerprint at 8 h was different from those of others using the no-overfitting OPLS-DA model (*R* ^2^ = 0.126, *Q* ^2^ = −0.29) (**Figure 5B**). The metabolites with VIP > 1 are listed in **Supplementary Figure S1**. Meanwhile, a correlation analysis was also employed for all logitudinal metabolic data (**Figure 5C**), and the results of heatmap clustering of the changed metabolites indicated that the disturbance at the 8-h time point was visibly different (**Figure 5D**).

Subsequently, comparison of the metabolic differences before and after sotorasib treatment (**Figure 5E**) demonstrated distinct segregation of all metabolites with the no-overfitting OPLS-DA model (*R* ^2^ = 0.475, *Q* ^2^ = −0.515) (**Figure 5F**). In addition, the hierarchical cluster correlation analysis showed that all metabolites presented obvious correlations due to sotorasib treatment (**Figure 5G**). Afterwards, calculation of the variable importance of the model (VIP) determined 20 metabolites with the highest VIP values (displayed in **Figure 5H**), such as amino acids (e.g., taurine, l-valine, and l-leucine) and carnitines (e.g., DL 10:0, DL 8:0-1, and DL 8:0-2).

**Figure 6:**
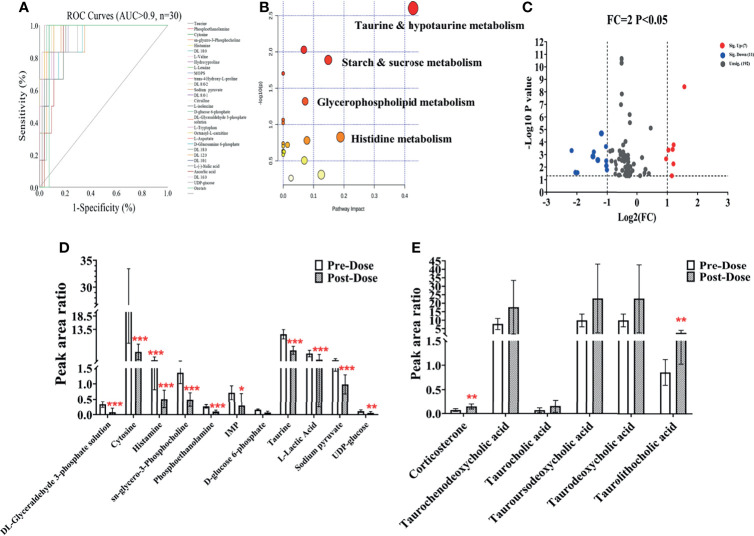
Metabolic changes after sotorasib treatment. **(A)** Receiver operating characteristic (ROC) curves of the metabolites administered sotorasib (AUC > 0.9, *n* = 30). **(B)** Overview of the pathway enrichment analysis of the altered metabolites between pre- and post-doses. **(C)** Volcano map of the metabolites with variable importance in the projections (VIP) >1 and fold change (FC) values >2 and <0.5 (*p* < 0.05). **(D, E)** Downregulated (*n* = 11) **(D)** and upregulated (*n* = 6) **(E)** metabolites compared with the pre-dose group. *p < 0.05; **p < 0.01; ***p < 0.001.

Considering the metabolic disturbance due to drug treatment, we further evaluated the transversal trajectories displayed in **Figure 6**. The results of the receiver operating characteristic (ROC) curve analysis demonstrated a total of 30 metabolites with AUC > 0.9 (listed in **Figure 6A**), which means that these potential biomarkers may be used for further investigation. After importing the raw data, 67 significantly changed metabolites were found between pre- and post-dose (**Supplementary Table S2**), and 19 metabolic pathways were disturbed, with the most significant pathway being taurine and hypotaurine metabolism (impact = 0.42857, *p* < 0.05) (**Figure 6B** and **Supplementary Table S3**). The volcano map of the FC results showed that a total of six metabolites were upregulated and 11 metabolites were downregulated after dosing (**Figure 6C**). Detailed information on the upregulated and downregulated metabolites is shown in **Figures 6D, E**. A reliable QC is indispensable for metabolomics. The PCA charts of the QC and unknown samples are shown in **Supplementary Figure S2**.

### 3.6 Correlation Analysis Between Metabolomics and Pharmacokinetics

**Figure 7:**
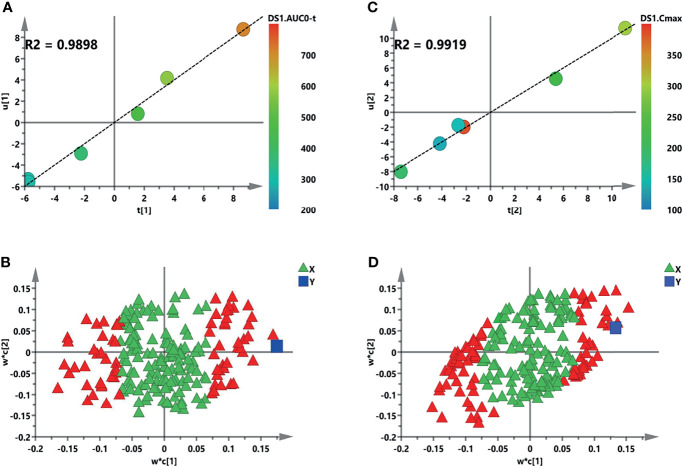
Initial partial least squares (PLS) models of the pre-dose metabolic profiles for predicting the pharmacokinetic (PK) parameters of sotorasib. **(A, B)** Score plots for the first latent variable of the AUC_0–_ *_t_* and *C*_max_ prediction models, respectively. Each *dot* represents a rat, plotted as the first latent variable (*X* block) *vs.* the AUC or *C*_max_ (*Y* block). *Color from blue to red* represents the response variable from low to high. **(C, D)** Loading plots for the AUC and *C*_max_ prediction models, respectively. The *blue box* represents the response variable. Each *triangle* represents a metabolite, and the *triangles in red* represent the metabolites with variable importance in the projections (VIP) >1.0.

For systemic investigation of the novel KRAS inhibitor of sotorasib, a modeled correlation analysis was performed using the PLS model. After examining the raw data, the metabolites clustered tightly in the PCA (**Supplementary Figure S3**). In the initial model, the two key PK parameters—AUC and *C*_max_—were adopted because of their predictive value for drug efficacy or toxicity (25). Thus, analysis of *X* (the predictive variable of 208 metabolic peak areas)–*Y* (the response variable of AUC or *C*_max_) was employed and modeled. A two-component PLS model was adopted to obtain a visible positive linear regression (*R* ^2^ = 0.9898 and *R* ^2^ = 0.9919) (**Figures 7A, C**, respectively). As described in this loading plot (**Figures 7B, D** **)**, the *X* variable on the top right or lower left corner represents positive or negative correlation to the AUC or *C*_max_, respectively. Moreover, a total of 73 (AUC) and 82 (*C*_max_) VIP > 1.0 *X* variables were recognized in view of the contribution of these variables to the PLS model (red triangles), which were chosen for subsequent prediction of the AUC and *C*_max_, respectively.

### 3.7 Prediction of AUC and *C*_max_ Based on Significant Metabolites

**Figure 8:**
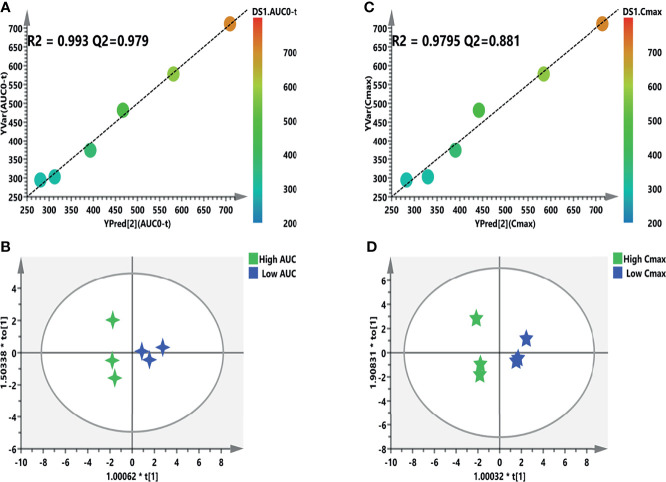
Refined models predicting the individualized pharmacokinetic (PK) parameters based on the screened biomarkers. **(A**, **C)** Regression plots of the predicted *vs.* the measured PK parameters (AUC or *C*_max_). *Color from blue to red* indicates the corresponding PK values from low to high. **(B**, **D)** Orthogonal projection to latent structures discriminant analysis (OPLS-DA) models discriminating the subgroups based on the screened biomarkers. *AUC*, area under the curve; *C*_max_, maximum concentration.

The individual PK profiles and key parameters are illustrated in **Supplementary Figure S4**. The FC values were 2.99 and 2.41 times. Pearson’s correlation analysis was used for the association between the PK parameters and VIP > 1.0 variables. Regarding the predictive model, six and nine VIP > 1.0 screened variables were significantly correlated with the AUC and *C*_max_, respectively (**Table 5**). Moreover, three common metabolites, namely, *N*,*N*-dimethylglycine, adenine, and OH-phenylpyruvate, were found in both predictive models. As illustrated in **Figure 8A**, a refined PLS model was constructed based on previous variables, which helped explain about 99.3% variation (*R* ^2^ *Y*) and predict 97.9% variation (*Q* ^2^) for AUC. Simultaneously, as shown in **Figure 8C**, it could explain about 97.95% variation (*R* ^2^ *Y*) and predict 88.1% variation (*Q* ^2^) in *C*_max_. The variables listed in **Table 5** were considered potential biomarkers for predicting the AUC or *C*_max_. To validate the predictive ability of the above screened potential biomarkers, the rats were divided into a high-value and a low-value group according to their AUC and *C*_max_. Discrimination based on the screened biomarkers was performed using OPLS-DA models. As described in **Figure 8B** (*R* ^2^ *X* = 0.876, *R* ^2^ *Y* = 0.907, *Q* ^2^ = 0.624) and **Figure 8D** (*R* ^2^ *X* = 0.988, *R* ^2^ *Y* = 0.974, *Q* ^2^ = 0.796), the selected six (AUC model) and nine (*C*_max_ model) metabolites completely distinguished the high- and low-value groups, which indicates that these potential biomarkers can potentially predict drug response or toxicity.

**Table 5:** Potential predictive biomarkers for the pharmacokinetics of sotorasib.

Model	Potential biomarker	VIP	Pearson’s coefficient
AUC refined model	*N*,*N*-dimethylglycine	2.44	0.955**
	Adenine	2.39	−0.934**
	OH-phenylpyruvate	2.24	−0.877*
	FA 20:1-iso1	2.17	−0.847*
	Glutaconylcarnitine/heptanoylcarnitine	2.15	−0.840*
	FA 20:0	2.13	−0.833*
*C*_max_ refined model	*N*,*N*-dimethylglycine	2.44	0.853*
	Adenine	2.39	−0.873*
	OH-phenylpyruvate	2.24	−0.970**
	2′-Deoxycytidine	1.98	0.977**
	FA 22:2	1.78	−0.890*
	d-Glucosamine 6-phosphate	1.72	−0.890*
	Thiamine hydrochloride (B1)	1.60	−0.821*
	l-Homoserine	1.44	0.871*
	FA 22:1	1.31	−0.835*

AUC, area under the curve; C_max_, maximum concentration; VIP, variable importance in the projections.

^*^p < 0.05; **p < 0.01.

## 4 Discussion

Despite a lot of efforts and works done to fight cancer worldwide, it has become one of the metabolic diseases, and the incidence and mortality rates are increasing year by year (1). Emerging evidence indicates that cancer is a metabolic disease, and metabolic reprogramming is one of the important hallmarks of cancer (26, 27). On May 28, 2021, sotorasib was granted accelerated approval by the US FDA for the treatment of advanced or metastatic non-small cell lung cancer (NSCLC) patients with the KRAS (G12C) mutation (8). Recently, integration analysis of PK and PM can not only provide a useful approach for the systemic investigation of therapeutic drugs but also reveal the internal pharmacological mechanism from the perspective of metabolite disturbance and drug exposure (28).

During the process of analytical method development, different chromatographic columns were examined and compared. Sotorasib showed a wide peak on the reversed-phase Symmetry C_18_ column (50 mm × 2.1 mm, 5 μm) using ACN as the organic phase. The broadening peak could be modified on the Waters XBrige C_18_ column (50 mm × 2.1 mm, 3.5 μm), and chromatography separation was accomplished with high efficiency. The shape of the peaks showed the best separation with better symmetry. MeOH and ACN were both evaluated, and the peak shape and response were greatly improved when using ACN. By carefully optimizing the composition and pH, the organic phase was ACN (A) and the aqueous phase was water (B). All mobile phases contained 0.1% formic acid. The column temperature was investigated at ambient temperature, 40°C, and at 55°C. Given the whole responses and the separation efficiency, 40°C and ambient temperature were set for the column temperature and the autosampler. Despite analogs or stable ILIS being better for the determination of analytes due to the analogous LC conditions, carbamazepine as the IS was suitable for the analyte, which satisfied this PK study.

The results of the method validation of the PK study, including selectivity, accuracy, precision, matrix effects, recovery, and stability, met the criteria of the guidelines of bioanalytical method validation (**Tables 1**–**3**). Retmana et al. (12) have recently reported on the PK of sotorasib following single oral administration of 20 mg/kg in mice. Upon administration, sotorasib exhibited rapid absorption, with *C*_max_ being achieved in <15 min. The other parameters were as follows: *T*_max_ = 0.21 ± 0.06 h, *C*_max_ = 4,231 ± 1,208 ng/ml, *t*_1/2_ = 0.60 ± 0.06 h, and AUC_0–4_ = 3,766 ± 896 ng·h/ml. Madhyastha et al. (11) compared the PK parameters after two administrations (intravenous *vs.* oral) at a dose of 50 mg/kg. With oral administration, the *t*_1/2_, *C*_max_, *T*_max_, and AUC_0–∞_ were 1.90 h, 123 ng/ml, 0.25 h, and 170 ng·h/ml, respectively.

The LLOQ of sotorasib was set at 0.5 ng/ml, with acceptable accuracy and precision, and the sensitivity was used to determine samples with low plasma concentrations. The results of the PK parameters were consistent with those in previous literature (11, 12). The robust and convenient HPLC-MS/MS method developed in this study had been applied successfully for PK, but further application is in the quantitation of sotorasib in human plasma. In our future works, we will continue to optimize the present assay and overcome this applicable limitation, which will undoubtedly benefit the therapeutic urgency of KRAS (G12C) inhibitors.

It is well known that sotorasib has broken the “non-druggable” curse and is the first KRAS-targeted drug approved by the FDA (10). PM is one of the most critical approaches to explore the mechanism, predict the drug response or toxicity, and optimize personalized drug dosage in order to improve efficacy and safety (18, 29–33). To date, no metabolic profile study has been published regarding the PM of sotorasib *in vivo* or *in vitro*. In this study, both longitudinal and transversal trajectories were assessed using multivariable statistics. In the longitudinal results, the characteristics of the 8-h metabolites were obviously different from those in other sampling time points, which indicates that the metabolic profile compared to the PK showed a delayed and an opposite trend. Additionally, the metabolic phenotype after sotorasib treatment was significantly segregated, and the metabolic pathways were also disturbed. Pan et al. (34) have recently published serum metabolomics in lung cancer-bearing mice after treatment with anlotinib. Compared with the control group, 13 differential metabolites and five potential metabolic pathways (i.e., glyoxylate and dicarboxylate metabolism; tryptophan metabolism; glycine, serine, and threonine metabolism; phenylalanine metabolism; and valine, leucine, and isoleucine biosynthesis) were observed. In this study, we found that the amino acids (e.g., l-valine, hydroxyproline, l-leucine, l-isoleucine, l-tryptophan, and l-aspartate), carnitines (e.g., DL 10:0, DL 8:0-2, DL 8:0-1, DL 16:0, DL 12:0, DL 10:1, and DL 18:0), and taurines were significantly changed upon sotorasib administration (**Supplementary Table S2**). Tryptophan plays a critical role in protein synthesis and is a precursor of bioactive compounds. It is an essential amino acid and regulator of antitumor immune response and cancer progression (35). Besides, the metabolism of tryptophan through the kynurenine pathway into free tryptophan has key functions in neurotransmission (36) and immune response regulation, and the accumulation of small-molecule inhibitors targeting tryptophan metabolism has been evaluated in the clinical phase (37). The branched-chain amino acids (BCAAs) such as l-valine, l-leucine, and l-isoleucine play crucial roles in the glucose or nutrition metabolism pathways [e.g., the mammalian target of rapamycin (mTOR) and phosphoinositide 3-kinase/protein kinase (PI3K/AKT) pathways] (38, 39). In this exploratory study, three common potential biomarkers—*N*,*N*-dimethylglycine (a derivative of the amino acid glycine), adenine, and OH-phenylpyruvate—were selected to predict drug exposure. As previously reported, reprogrammed BCAA metabolism can directly promote cancer progression and development (40). Besides, Ni et al. (41) reported that six metabolites (glycine, valine, methionine, citrulline, arginine, and C16 carnitine), using the PLS-DA model, were indicated to distinguish lung cancer from healthy controls and may be screening biomarkers for patients with lung cancer. Glycine or its derivative may be used for diagnosing lung cancer or real-time monitoring/predicting the efficacy of sotorasib. It has been reported that cholic acid or bile acid plays a pivotal role in the process of traditional Chinese medicine for the treatment of malignant tumors. A previous study demonstrated that bile acid metabolism, such as taurine, chenodeoxycholic acid, cholic acid, and deoxycholic acid, was regulated by mirabilite in colorectal cancer, and the results of this study—on the disturbance of metabolites before and after sotorasib administration—were basically consistent with those in published literature on the treatment of colorectal cancer (42).

Since the first description by Yoon et al. for the immunosuppressive drug tacrolimus, PM has been increasingly applied to predict the drug response, safety/toxicity, and the PK profile (43). After checking for outliers, a two-stage PLS analysis including an initial and a refined model was constructed between the metabolites and PK parameters (AUC and *C*_max_). Finally, a total of six and nine metabolites were selected and further validated to further verify the predictive efficiency (**Figure 8**). Although this study used a limited number of samples, several metabolite biomarkers were firstly discovered and were utilized to predict drug exposure/toxicity. Amino acids, carnitines, adenine, and fatty acids are important metabolites in metabolomics and have recently attracted the attention of an increasing number of cancer research studies. Oncogenic KRAS can activate the ACLY enzyme to promote the conversion of citrate to acetyl-CoA and enhance *de novo* fatty acid biosynthesis (44). Gene expression and metabolic flux analyses have shown that mutant KRAS upregulates the expression of the GLUT1 glucose transporter to promote glucose uptake, as well as inducing the expressions of the rate-limiting enzymes of glycolysis (hexokinases 1 and 2) or other key glycolytic enzymes (e.g., PFK1, ENO1, and LDHA), which enhances the glycolytic flux and promotes the production of intermediates (e.g., glucose-6-P and fructose-6-P) (45). Although substantial progress has been made in the interplay between KRAS mutation and metabolic reprogramming, a lot of work is still needed to fully discover the crosstalk of sotorasib from other aspects besides the PK and PM.

To date, no investigation has been reported regarding the comprehensive metabolic fingerprint after sotorasib treatment and the correlation with the PK parameters. Indeed, several limitations should be mentioned. Firstly, although the recovery and matrix effect criteria were not defined in the guidelines for quantitative biological sample determination, there is much opportunity for improvement in the biological sample pretreatment methods of plasma in this PK study. Secondly, the sample size of this exploratory research was small, and more data and external validation may be needed to pool and verify these results. Thirdly, due to the non-availability of blood samples from cancer patients, we only investigated the metabolomic profile in rats after a single administration of sotorasib. A more in-depth understanding of these concerns will pave the way for the development of well-tolerated and effective therapies for patients with KRAS-driven cancer. Taken together, our work is the first to reveal the comprehensive metabolite trajectories induced by sotorasib and the predictive drug exposure/toxicity biomarkers in rats, which will provide a notable scientific contribution to prevention or therapy in patients with the KRAS (G12C) mutation.

## 5 Conclusion

A robust and sensitive HPLC-MS/MS assay was firstly developed and fully validated for the quantitation of sotorasib in rat plasma. Both longitudinal and transversal metabolism characteristics were revealed systemically. After treatment with sotorasib, 19 metabolic pathways were disturbed, and the most significant pathway was taurine and hypotaurine metabolism. A total of six metabolites were upregulated and 11 metabolites were downregulated after dosing when the FC setting was 2. Moreover, an integrated PM and PK analysis was employed to predict the metabolic phenotype and drug exposure of sotorasib. Using two-stage PLS and OPLS-DA models, six and nine screened variables were significantly correlated with the AUC and *C*_max_ of sotorasib, respectively, and the potential predictive biomarkers could discriminate between the high- and low-exposure groups with reliable model validation. Collectively, our work is the first to reveal the comprehensive metabolite trajectories induced by sotorasib and to investigate biomarkers for predicting drug exposure/toxicity, which will provide a notable scientific contribution to prevention or therapy in patients with the KRAS (G12C) mutation.

## Data Availability Statement

The original contributions presented in the study are included in the article/**Supplementary Material**. Further inquiries can be directed to the corresponding author.

## Ethics Statement

The animal study was reviewed and approved by the Animal Care and Ethics Committee of Beijing Chao-Yang Hospital, Capital Medical University.

## Author Contributions

PD: conceptualization, investigation, and writing—original draft. TH: methodology. TH and ZA: writing—review and editing. LL: resources and supervision. All authors contributed to the article and approved the submitted version.

## Conflict of Interest

The authors declare that the research was conducted in the absence of any commercial or financial relationships that could be construed as a potential conflict of interest.

## Publisher’s Note

All claims expressed in this article are solely those of the authors and do not necessarily represent those of their affiliated organizations, or those of the publisher, the editors and the reviewers. Any product that may be evaluated in this article, or claim that may be made by its manufacturer, is not guaranteed or endorsed by the publisher.

## References

[1] Sung H Ferlay J Siegel RL Laversanne M Soerjomataram I Jemal A. Global Cancer Statistics 2020: GLOBOCAN Estimates of Incidence and Mortality Worldwide for 36 Cancers in 185 Countries. CA Cancer J Clin (2021) 71(3):209–49. doi: 10.3322/caac.21660 33538338

[2] Mustachio LM Chelariu-Raicu A Szekvolgyi L Roszik J. Targeting KRAS in Cancer: Promising Therapeutic Strategies. Cancers (2021) 13(6):1204. doi: 10.3390/cancers13061204 33801965 PMC7999304

[3] Kim D Xue JY Lito P. Targeting KRAS (G12C): From Inhibitory Mechanism to Modulation of Antitumor Effects in Patients. Cell (2020) 183(4):850–9. doi: 10.1016/j.cell.2020.09.044 PMC7669705 33065029

[4] Prior IA Lewis PD Mattos C. A Comprehensive Survey of Ras Mutations in Cancer. Cancer Res (2012) 72(10):2457–67. doi: 10.1158/0008-5472.CAN-11-2612 PMC3354961 22589270

[5] Cox AD Fesik SW Kimmelman AC Luo J Der CJ. Drugging the Undruggable RAS: Mission Possible? Nat Rev Drug Discov (2014) 13(11):828–51. doi: 10.1038/nrd4389 PMC4355017 25323927

[6] Canon J Rex K Saiki AY Mohr C Cooke K Bagal D. The Clinical KRAS (G12C) Inhibitor AMG 510 Drives Anti-Tumour Immunity. Nature (2019) 575(7781):217–23. doi: 10.1038/s41586-019-1694-1 31666701

[7] Govindan R Fakih M Price T Falchook G Desai J Kuo J. Phase I Study of AMG 510, A Novel Molecule Targeting KRAS G12C Mutant Solid Tumours. Ann Oncol (2019) 30:v163–4. doi: 10.1093/annonc/mdz244.008

[8] Blair HA. Sotorasib: First Approval. Drugs (2021) 81(13):1573–9. doi: 10.1007/s40265-021-01574-2 PMC8531079 34357500

[9] Fakih M Desai J Kuboki Y Strickler JH Price TJ Durm GA. Codebreak 100: Activity of AMG 510, a Novel Small Molecule Inhibitor of KRASG12C, in Patients With Advanced Colorectal Cancer. J Clin Oncol (2020) 38(15_suppl):4018. doi: 10.1200/JCO.2020.38.15_suppl.4018

[10] Uprety D Adjei AA. KRAS: From Undruggable to a Druggable Cancer Target. Cancer Treat Rev (2020) 89:102070. doi: 10.1016/j.ctrv.2020.102070 32711246

[11] Madhyastha N Samantha SK Dittakavi S Markose M Mallurwar SR Zainuddin M. Validated HPLC-MS/MS Method for Quantitation of AMG 510, a KRAS G12C Inhibitor, in Mouse Plasma and Its Application to a Pharmacokinetic Study in Mice. BioMed Chromatogr (2021) 35(4):e5043. doi: 10.1002/bmc.5043 33283304

[12] Retmana IA Loos NH Schinkel AH Beijnen JH Sparidans RW. Quantification of KRAS Inhibitor Sotorasib in Mouse Plasma and Tissue Homogenates Using Liquid Chromatography-Tandem Mass Spectrometry. J Chromatogr B (2021) 1174:122718. doi: 10.1016/j.jchromb.2021.122718 33957355

[13] Schmidt DR Patel R Kirsch DG Lewis CA Vander Heiden MG Locasale JW. Metabolomics in Cancer Research and Emerging Applications in Clinical Oncology. CA: Cancer J Clin (2021) 0:1–26. doi: 10.3322/caac.21670 PMC8298088 33982817

[14] Clayton TA Lindon JC Cloarec O Antti H Charuel C Hanton G. Pharmaco-Metabonomic Phenotyping and Personalized Drug Treatment. Nature (2006) 440(7087):1073–7. doi: 10.1038/nature04648 16625200

[15] Thomas T Stefanoni D Reisz JA Nemkov T Bertolone L Francis RO. COVID-19 Infection Results in Alterations of the Kynurenine Pathway and Fatty Acid Metabolism That Correlate With IL-6 Levels and Renal Status. JCI Insight (2020) 5(14):e140327. doi: 10.1172/jci.insight.140327 PMC7453907 32559180

[16] Kaddurah-Daouk R Weinshilboum R Network PR. Metabolomic Signatures for Drug Response Phenotypes: Pharmacometabolomics Enables Precision Medicine. Clin Pharmacol Ther (2015) 98(1):71–5. doi: 10.1002/cpt.134 PMC4938244 25871646

[17] Hu T Shi C Liu L Li P Sun Y An Z. A Single-Injection Targeted Metabolomics Profiling Method for Determination of Biomarkers to Reflect Tripterygium Glycosides Efficacy and Toxicity. Toxicol Appl Pharmacol (2020) 389:114880. doi: 10.1016/j.taap.2020.114880 31945383

[18] Hu T An Z Sun Y Wang X Du P Li P. Longitudinal Pharmacometabonomics for Predicting Malignant Tumor Patient Responses to Anlotinib Therapy: Phenotype, Efficacy and Toxicity. Front Oncol (2020) 10:2373. doi: 10.3389/fonc.2020.548300 PMC7689013 33282726

[19] Du P Guan Y An Z Li P Liu L. A Selective and Robust UPLC-MS/MS Method for the Simultaneous Quantitative Determination of Anlotinib, Ceritinib and Ibrutinib in Rat Plasma and Its Application to a Pharmacokinetic Study. Analyst (2019) 144(18):5462–71. doi: 10.1039/C9AN00861F 31380858

[20] Evaluation, Research CFD. Bioanalytical Method Validation Guidance For Industry. Center for Drug Evaluation and Research of the U.S. Maryland: Department of Health and Human Services Food and Drug Administration. (2018). Available at: https://www.fda.gov/downloads/Drugs GuidanceComplianceRegulatoryInformation/Guidances/UCM070107.pdf. 2018updated 2018-01-01%\ 2020-05-11 11:19:00

[21] Chinese PC. The Pharmacopoeia of the People’s Republic of China, 2015 Edition Part IV 2015 2015-01-01%\ 2020-03-16 20:55:00. Beijing (2015)

[22] Du P Li P Zhao R Liu H Liu L. Optimized UPLC-MS/MS Method for the Quantitation of Olanzapine in Human Plasma: Application to a Bioequivalence Study. Bioanalysis (2019) 11(13):1291–302. doi: 10.4155/bio-2019-0114 31379195

[23] Meyer VR. Measurement Uncertainty. J Chromatogr AData Anal Chromatogr (2007) 1158(1–2):15–24. doi: 10.1016/j.chroma.2007.02.082 17359984

[24] Du P Wang G Yang S Li P Liu L. Quantitative HPLC-MS/MS Determination of Nuc, the Active Metabolite of Remdesivir, and Its Pharmacokinetics in Rat. Anal Bioanal Chem (2021) 413(23):5811–20. doi: 10.1007/s00216-021-03561-8 PMC8302467 34302183

[25] Xing X Ma P Huang Q Qi X Zou B Wei J. Predicting Pharmacokinetics Variation of Faropenem Using a Pharmacometabonomic Approach. J Proteome Res (2019) 19(1):119–28. doi: 10.1021/acs.jproteome.9b00436 31617722

[26] Seyfried TN Shelton LM. Cancer as a Metabolic Disease. Nutr Metab (2010) 7(1):1–22. doi: 10.1186/1743-7075-7-7 PMC2845135 20181022

[27] Hanahan D Weinberg RA. Hallmarks of Cancer: The Next Generation. Cell (2011) 144(5):646–74. doi: 10.1016/j.cell.2011.02.013 21376230

[28] Varshavi D Varshavi D Everett JR. The Role of Pharmacometabonomics in Predicting Drug Pharmacokinetics. Future Sci (2016) 1(1):5–8. doi: 10.4155/ipk-2016-0010

[29] An Z Wang X Li P He J Liu L. Exploring the Metabolic Characteristics and Pharmacokinetic Variation of Paroxetine in Healthy Volunteers Using a Pharmacometabonomic Approach. J Pharm Biomed Anal (2021) 204:114224. doi: 10.1016/j.jpba.2021.114224 34265484

[30] Du P Wang G Zhao R Hu T Li H An Z. Eicosanoids Metabolomic Profile of Remdesivir Treatment in Rat Plasma by High-Performance Liquid Chromatography Mass Spectrometry. Front Pharmacol (2021) 12:747450. doi: 10.3389/fphar.2021.747450 PMC8511316 34658883

[31] Mussap M Loddo C Fanni C Fanos V. Metabolomics in Pharmacology-A Delve Into the Novel Field of Pharmacometabolomics. Expert Rev Clin Pharmacol (2020) 13(2):115–34. doi: 10.1080/17512433.2020.1713750 31958027

[32] Du P Hu T An Z Li P Liu L. Simultaneous Quantitative Determination of Arachidonic Acid and Cascade Metabolites in Rat Serum by UPLC-MS/MS: Application for Longitudinal Metabolomics of Anlotinib. Analyst (2020) 145(14):4972–81. doi: 10.1039/D0AN00867B 32515434

[33] Nicholson JK Everett JR Lindon JC. Longitudinal Pharmacometabonomics for Predicting Patient Responses to Therapy: Drug Metabolism, Toxicity and Efficacy. Expert Opin Drug Metab Toxicol (2012) 8(2):135–9. doi: 10.1517/17425255.2012.646987 22248264

[34] Pan X Chen W Nie M Liu Y Xiao Z Zhang Y. A Serum Metabolomic Study Reveals Changes in Metabolites During the Treatment of Lung Cancer-Bearing Mice With Anlotinib. Cancer Manage Res (2021) 13:6055. doi: 10.2147/CMAR.S300897 PMC8349534 34377024

[35] Opitz CA Patterson LFS Mohapatra SR Dewi DL Sadik A Platten M. The Therapeutic Potential of Targeting Tryptophan Catabolism in Cancer. Br J Cancer (2020) 122(1):30–44. doi: 10.1038/s41416-019-0664-6 31819194 PMC6964670

[36] Platten M Nollen EA Röhrig UF Fallarino F Opitz CA. Tryptophan Metabolism as a Common Therapeutic Target in Cancer, Neurodegeneration and Beyond. Nat Rev Drug Discov (2019) 18(5):379–401. doi: 10.1038/s41573-019-0016-5 30760888

[37] Cheong JE Ekkati A Sun L. A Patent Review of IDO1 Inhibitors for Cancer. Expert Opin Ther Patents (2018) 28(4):317–30. doi: 10.1080/13543776.2018.1441290 29473428

[38] Lynch CJ Adams SH. Branched-Chain Amino Acids in Metabolic Signalling and Insulin Resistance. Nat Rev Endocrinol (2014) 10(12):723–36. doi: 10.1038/nrendo.2014.171 PMC4424797 25287287

[39] Jewell JL Russell RC Guan K-L. Amino Acid Signalling Upstream of Mtor. Nat Rev Mol Cell Biol (2013) 14(3):133–9. doi: 10.1038/nrm3522 PMC3988467 23361334

[40] Hattori A Tsunoda M Konuma T Kobayashi M Nagy T Glushka J. Cancer Progression by Reprogrammed BCAA Metabolism in Myeloid Leukaemia. Nature (2017) 545(7655):500–4. doi: 10.1038/nature22314 PMC5554449 28514443

[41] Ni J Xu L Li W Zheng C Wu L. Targeted Metabolomics for Serum Amino Acids and Acylcarnitines in Patients With Lung Cancer. Exp Ther Med (2019) 18(1):188–98. doi: 10.3892/etm.2019.7533 PMC6566041 31258653

[42] Sun H Zhang H-l Zhang A-h Zhou X-h Wang X-q Han Y. Network Pharmacology Combined With Functional Metabolomics Discover Bile Acid Metabolism as a Promising Target for Mirabilite Against Colorectal Cancer. RSC Adv (2018) 8(53):30061–70. doi: 10.1039/C8RA04886J PMC9085400 35546810

[43] Phapale P Kim SD Lee H Lim M Kale D Kim YL. An Integrative Approach for Identifying a Metabolic Phenotype Predictive of Individualized Pharmacokinetics of Tacrolimus. Clin Pharmacol Ther (2010) 87(4):426–36. doi: 10.1038/clpt.2009.296 20182421

[44] Carrer A Trefely S Zhao S Campbell SL Norgard RJ Schultz KC. Acetyl-Coa Metabolism Supports Multistep Pancreatic Tumorigenesis. Cancer Discov (2019) 9(3):416–35. doi: 10.1158/2159-8290.CD-18-0567 PMC6643997 30626590

[45] Mukhopadhyay S Vander Heiden MG McCormick F. The Metabolic Landscape of RAS-Driven Cancers From Biology to Therapy. Nat Cancer (2021) 2(3):271–83. doi: 10.1038/s43018-021-00184-x PMC8045781 33870211

